# Supplementary material for: A Comprehensive and Integrative Approach to MeCP2 Disease Transcriptomics
Source: Int J Mol Sci. 2023 Mar 7;24(6):5122. doi: 10.3390/ijms24065122 (PMC10049497; doi:10.3390/ijms24065122)
Supplement: Supplementary file 1 [file ijms-24-05122-s001.zip › ijms-2192040-supplementary/Supplementary Figures.pdf]

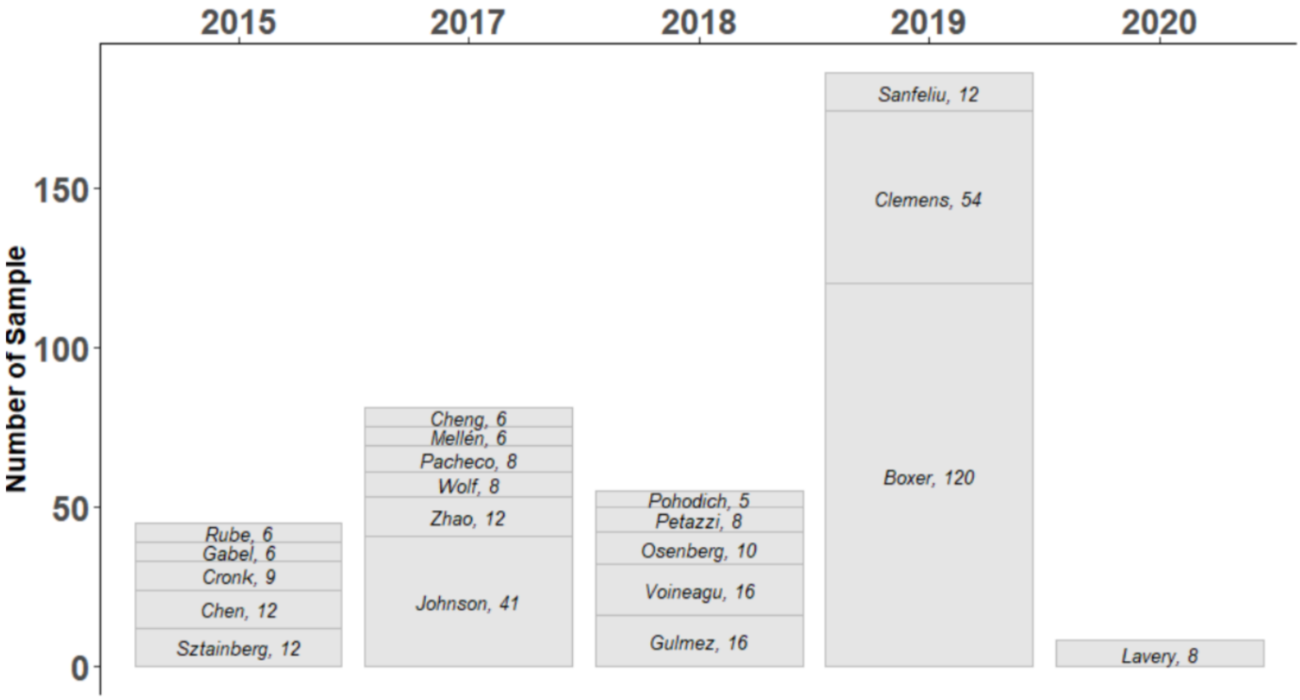

Figure S1. Mouse sample number. Bar plot of number of mouse samples by year and first author.

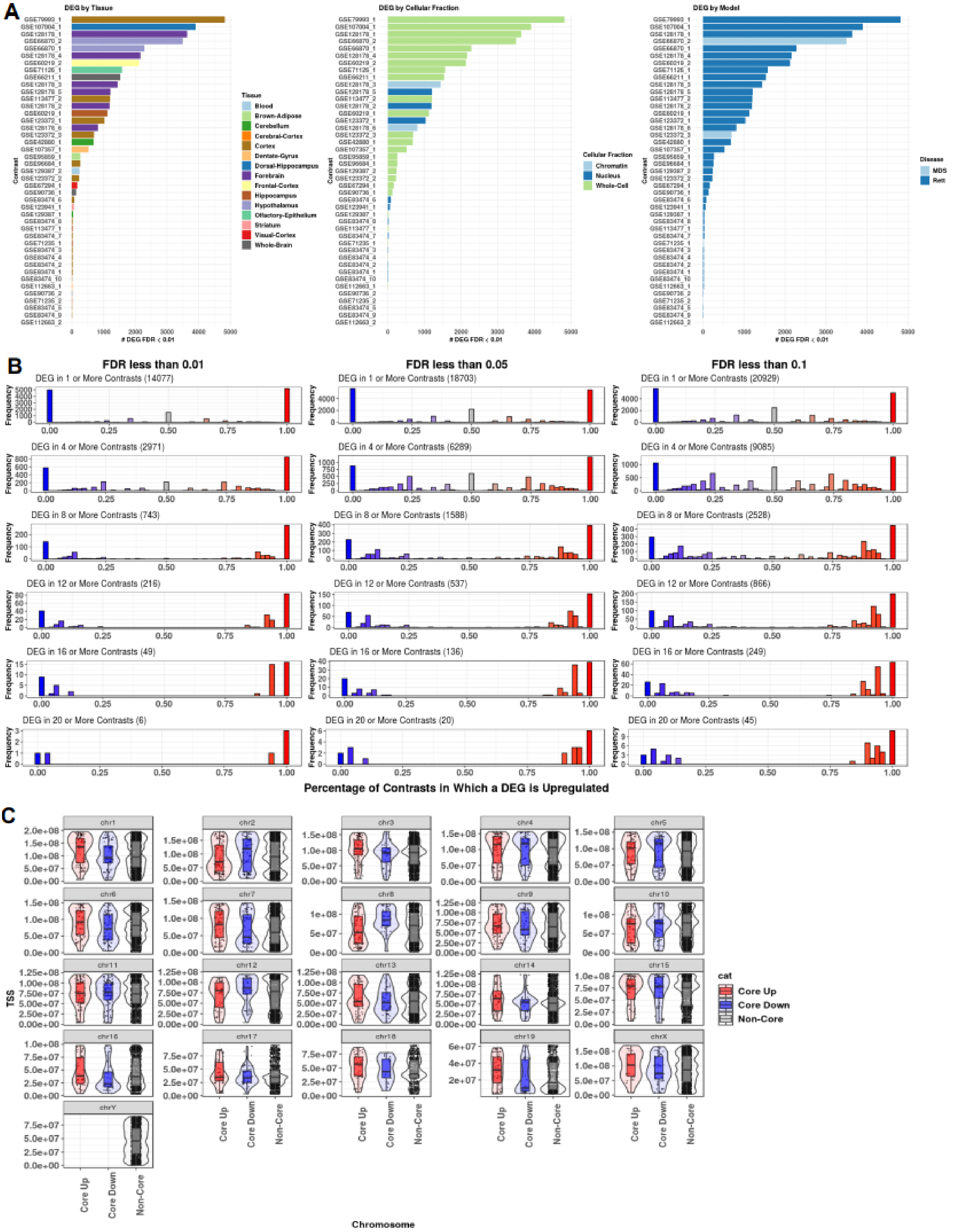

Figure S2. Expanded examination of common core characteristics.(A). Bar plots of number of DEGs from each contrast, colored and annotated by tissue, cell fraction, and model. No strong bias

is observed for any of these characteristics by DEG number.(B). Histograms of significantly up- and downregulated genes cut for different FDR thresholds and the number of total contrasts in which a DEG appears. Genes at the extreme ratios of 0 or 1 for percent upregulated are highly concordant across contrasts, whereas genes that fall into the middle are discordant. For consistency in this analysis, we inverted the direction of fold change for the four contrasts of TG model. (C). Exploration of genome location trends in the common core. Box plot, violin plot, and jittered dots are plotted for genome coordinate of TSS for each core DEG. Chromosome 8 was selected for further analysis, with random baseline genes added for detection of robust trends.

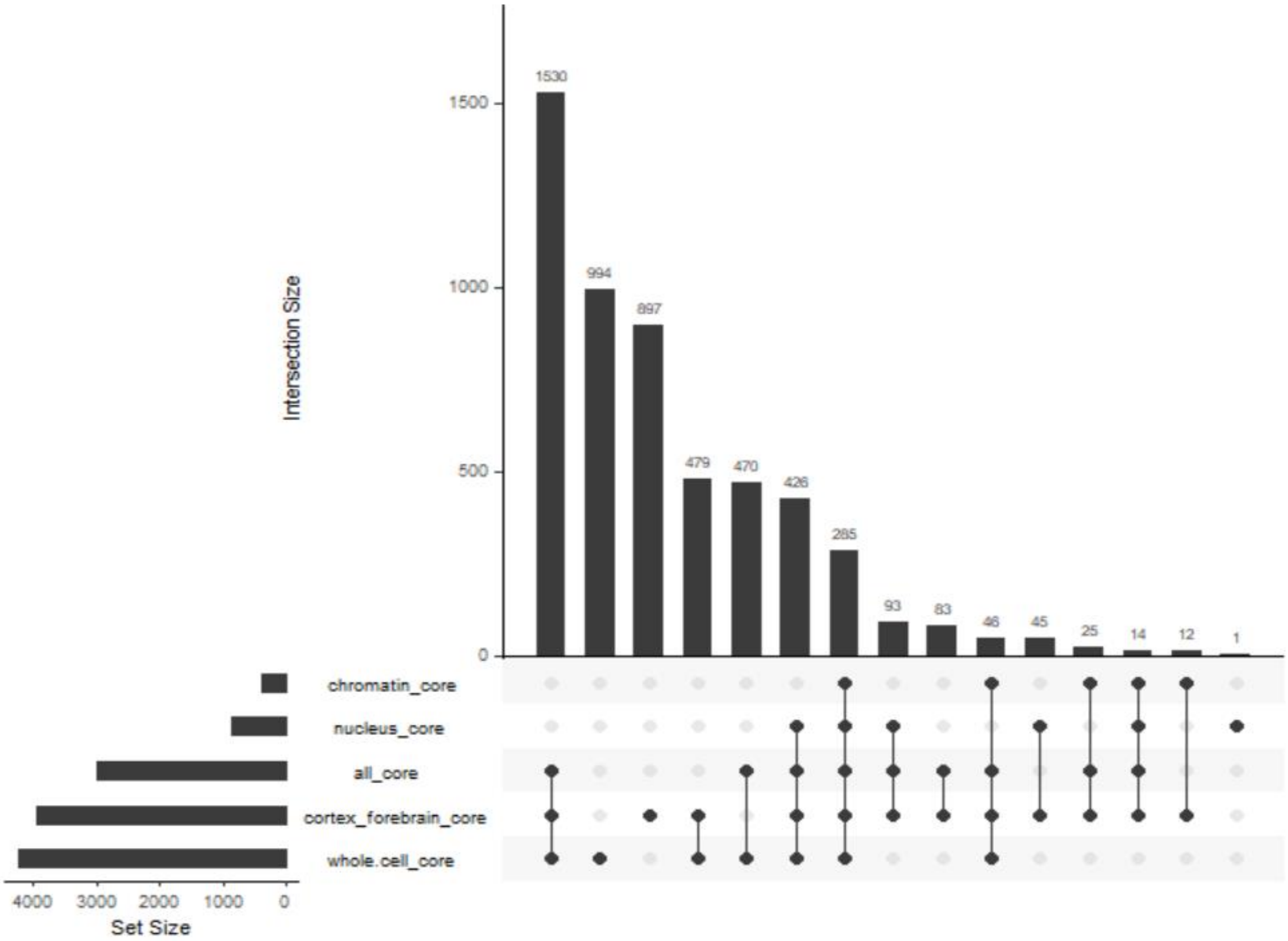

Figure S3. Overlap between specific common core designations. Upset plot to examine overlap between alternative core designations (chromatin, nucleus, whole cell, cortex-forebrain) and the common core. There is some overlap with all cores, demonstrating the absence of a strong bias in the types of data that contribute genes to the common core.

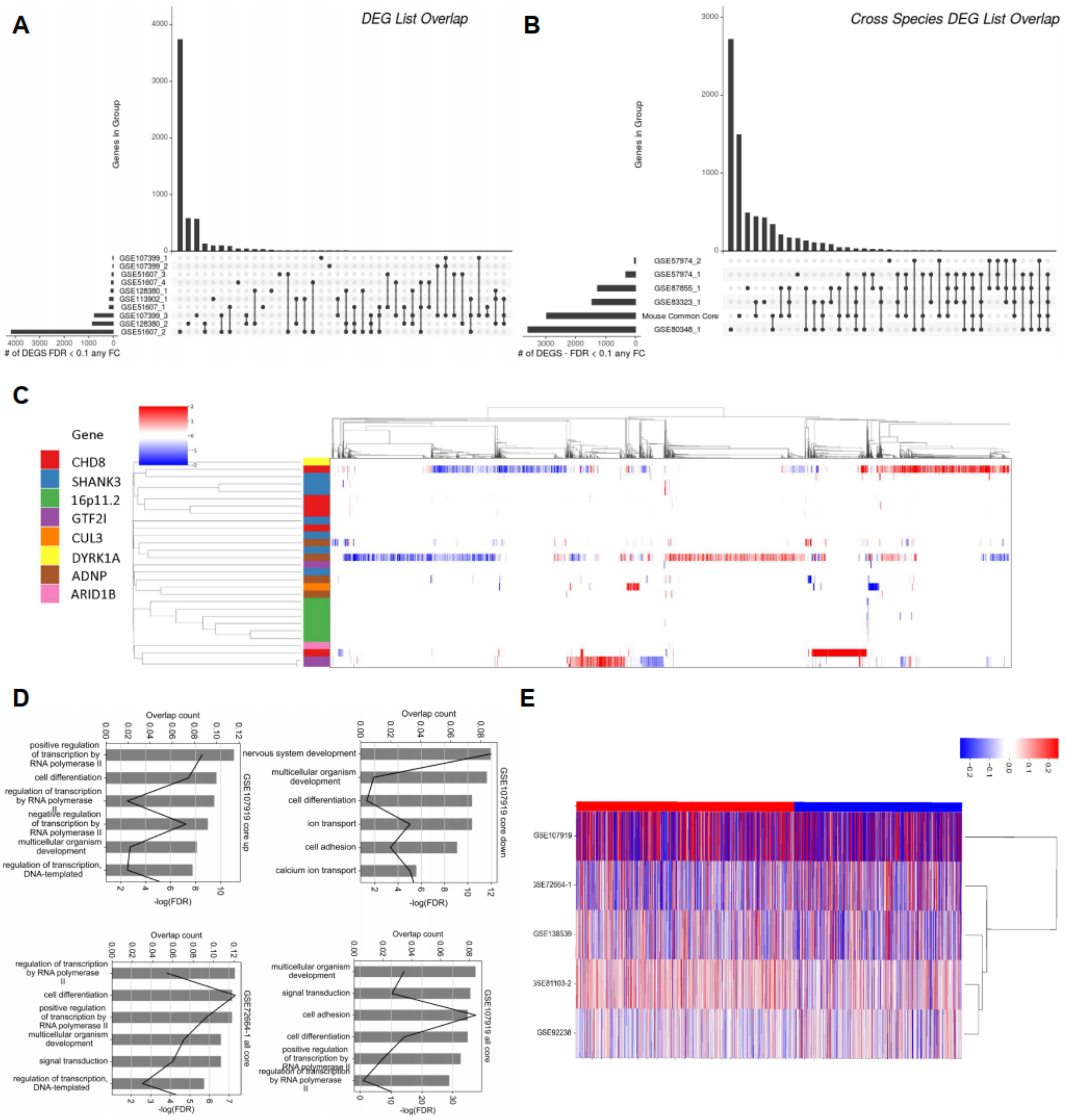

Figure S4. Mouse transcriptome translation to other models (A). Upset plot to examine overlap between the DEG lists from human contrasts. **(**B). Upset plot to examine overlap between the DEG lists from other species contrasts.**(**C). Heatmap of genes (rows) by contrasts (columns). Contrasts are labelled by ASD model gene. **(**D). Gene ontology enrichment on specific overlapping gene sets. Bar length represents the proportion of genes enriched with the term in the cluster and the line plot represents the FDR of the enrichment. **(E)**. Heatmaps plotted to compare the direction of dysregulation to the consensus from mouse data. Genes examined are the mouse common core, and plots are annotated on mouse common down and mouse common up. Only ASD contrasts with significant overlap to MeCP2 core are included.

A

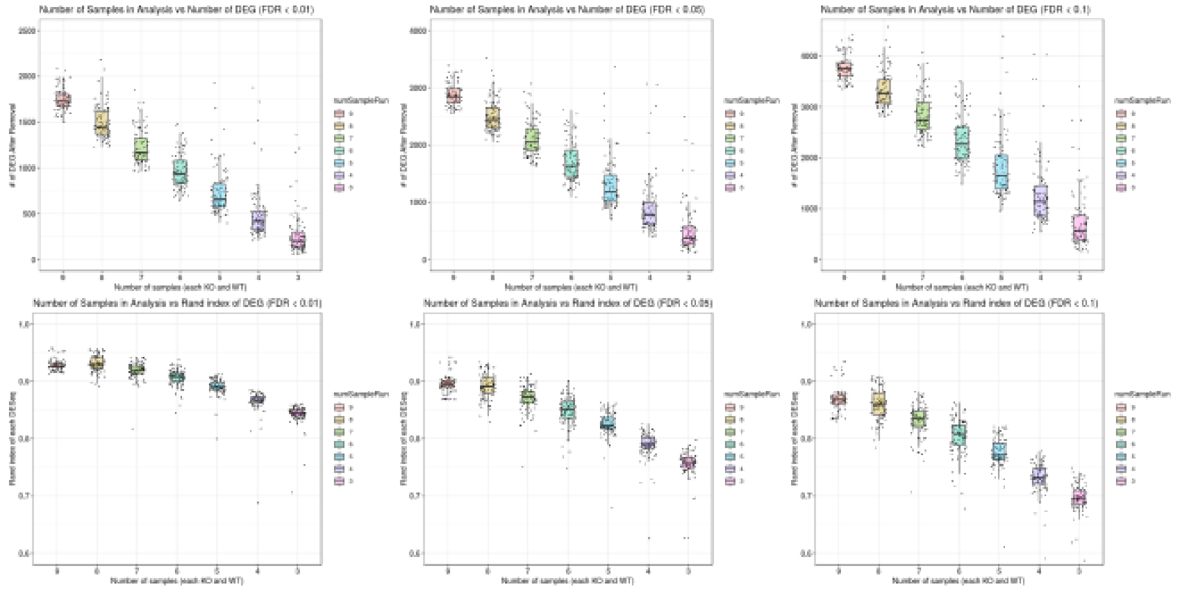
Number of Samples in Analysis vs Number of DEG (FDR < 0.01)
Number of Samples in Analysis vs Number of DEG (FDR < 0.05)
Number of Samples in Analysis vs Number of DEG (FDR < 0.1)
Number of Samples in Analysis vs Rand index of DEG (FDR < 0.01)
Number of Samples in Analysis vs Rand index of DEG (FDR < 0.05)
Number of Samples in Analysis vs Rand index of DEG (FDR < 0.1)
Number of samples (each KO and WT)
numSampleRun

B

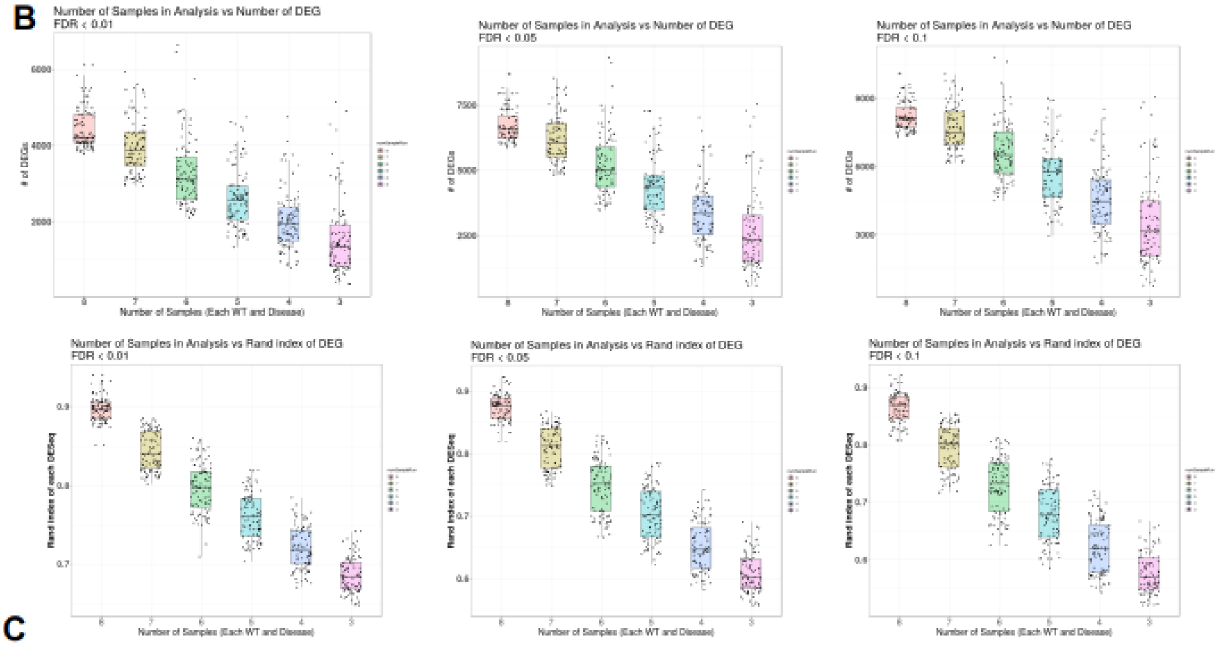
Number of Samples in Analysis vs Number of DEG
FDR < 0.01
Number of Samples in Analysis vs Number of DEG
FDR < 0.05
Number of Samples in Analysis vs Number of DEG
FDR < 0.1
Number of Samples in Analysis vs Rand index of DEG
FDR < 0.01
Number of Samples in Analysis vs Rand index of DEG
FDR < 0.05
Number of Samples in Analysis vs Rand index of DEG
FDR < 0.1
# of DEGs
Rand index of each DESeq
Number of Samples (Each WT and Disease)

C

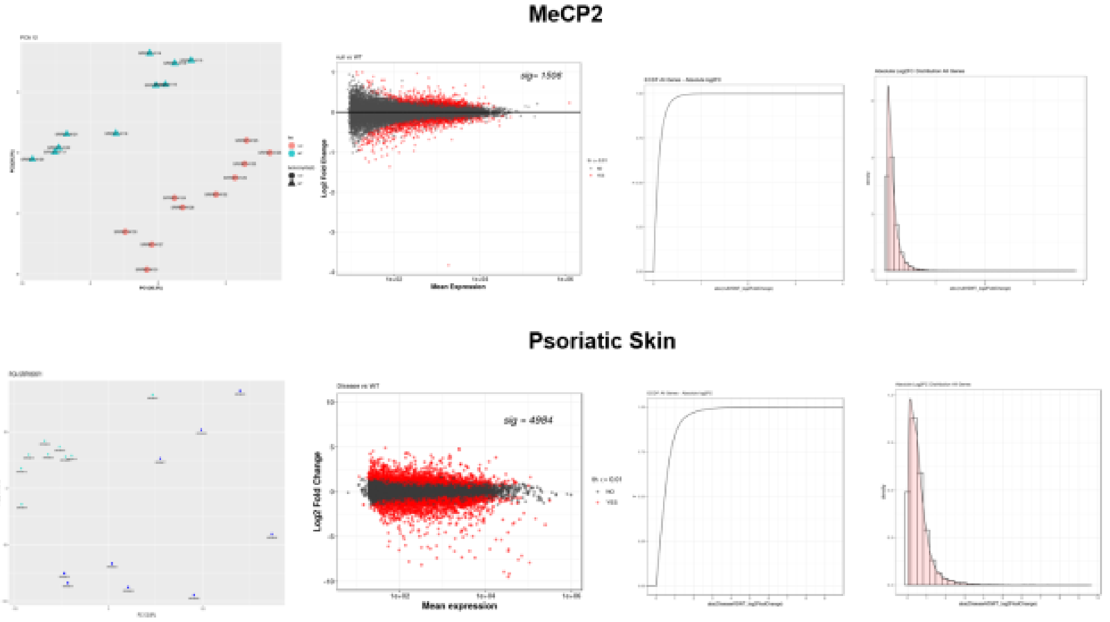
MeCP2
sig= 1506
Log2 Fold Change
Mean Expression
Psoriatic Skin
sig = 4984
Log2 Fold Change
Mean expression

Figure S5. Downsampling analysis (A) DEG analysis run on subsets of n = 10 from the MeCP2 data set. Cutoffs are sample numbers 9 through 3. Each cutoff number was repeated 100 times, with random samples discarded each time. Downsampled MeCP2 data at FDR thresholds < 0.01, 0.05, and 0.1. Rand index calculated for each downsampled result against full (n = 10) sample space. **(**B) DEG analysis run on subsets of n = 9 psoriatic skin data set. Cutoffs are sample numbers 8 through 3. Each cutoff number was repeated 100 times, with random samples discarded each time. Downsampled MeCP2 data at FDR thresholds < 0.01, 0.05, and 0.1. Rand index calculated for each downsampled result against full (n = 9) sample space. (C). PCA, MAplot, eCDF, and distribution of fold change for both MeCP2 and psoriatic skin data.

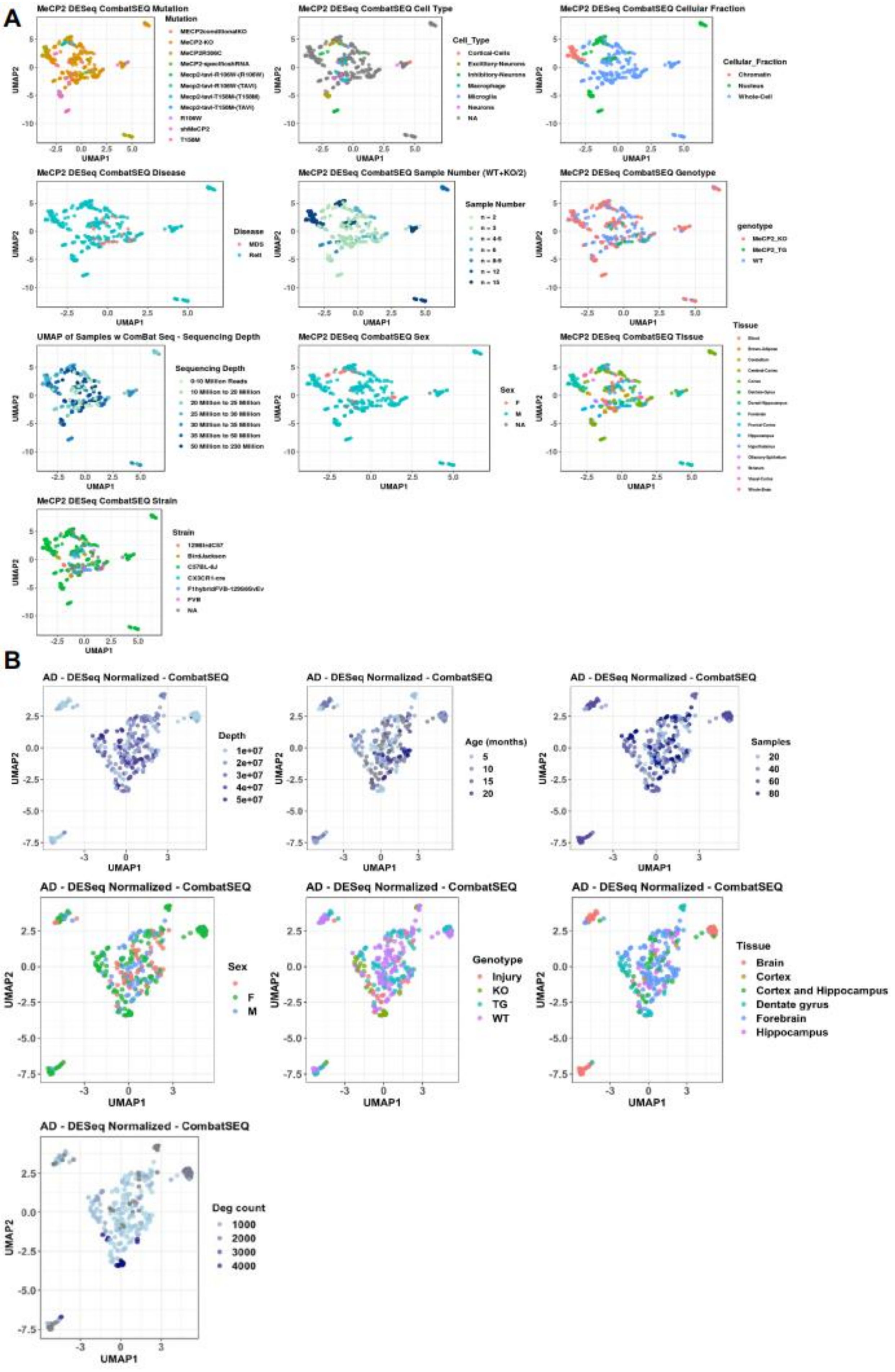
A
MeCP2 DESeq CombatSEQ Mutation
MeCP2 DESeq CombatSEQ Cell Type
MeCP2 DESeq CombatSEQ Cellular Fraction
MeCP2 DESeq CombatSEQ Disease
MeCP2 DESeq CombatSEQ Sample Number (WT+KO/2)
MeCP2 DESeq CombatSEQ Genotype
UMAP of Samples w ComBat Seq - Sequencing Depth
MeCP2 DESeq CombatSEQ Sex
MeCP2 DESeq CombatSEQ Tissue
MeCP2 DESeq CombatSEQ Strain
B
AD - DESeq Normalized - CombatSEQ
Depth
1e+07
2e+07
3e+07
4e+07
5e+07
Age (months)
5
10
15
20
Samples
20
40
60
80
Sex
F
M
Genotype
Injury
KO
TG
WT
Tissue
Brain
Cortex
Cortex and Hippocampus
Dentate gyrus
Forebrain
Hippocampus
Deg count
1000
2000
3000
4000
UMAP1
UMAP2

Figure S6. Batch effect UMAP visualization (A). UMAP on normalized and batch-corrected data from MeCP2 mouse data, colored for meta-characteristics. Some show good clustering on the characteristic (cell fraction) and some do not (sequencing depth). (**B**). UMAP on normalized and batch-corrected on Alzheimer's data, colored for meta-characteristics.

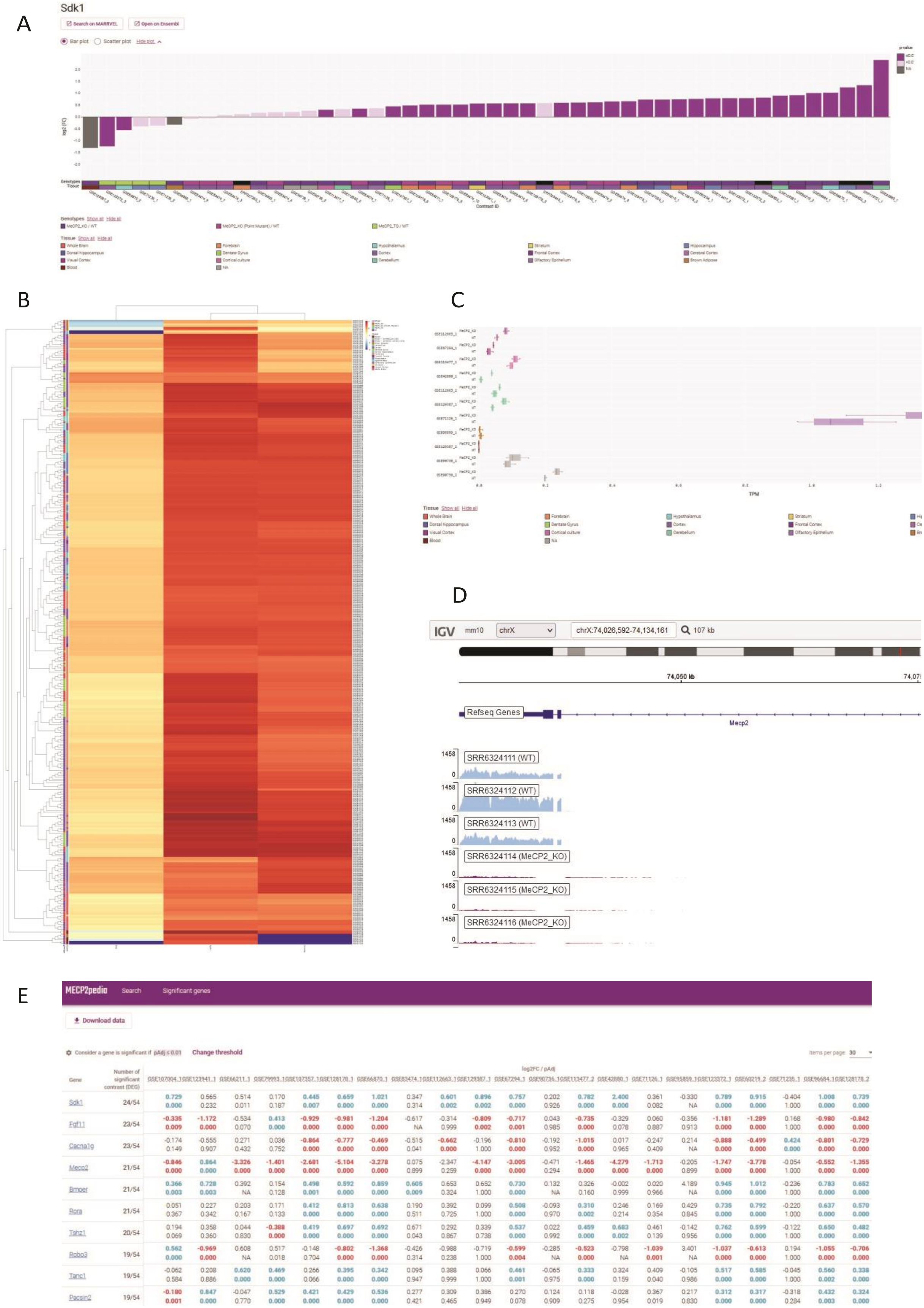

Figure S7. Portal demonstration. (**A**). Bar plot to compare one gene (Sdk1) across studies. Sdk1 is the top core gene by number of significant contrasts. Expression follows a clear general trend of up in Rett models, down in Mecp2 duplication syndrome models. Built in links to MARRVEL and Ensembl make follow up literature searches and biological understanding a seamless process. This can be filtered by tissue(s) and disease model of interest. (**B**). Example heatmap result from multi-gene search using the top 3 core genes, Sdk1, Fgf11, and Cacna1g. (**C**). TPM plot example showing a cross study expression comparison of Sdk1. This can be filtered by tissue(s) of interest. (**D**). IGV genomic tracks at example zoomed to Mecp2 showing a 3v3 knockout data set. Any 1 or 2 studies can be loaded at the same time to compare. (**E**). Significant genes tab accessible from the top panel of the portal, all summarized differential expression results are downloadable as a table. The data is filterable on p-adjusted and log2 fold change. The default gene order is based on the same criteria as our core genes – number of significant contrasts defined by p-adjusted < 0.01. Therefore, our top individual gene findings are conveniently presented here.
